# Risk Factors for the Occurrence of Femoral Fractures in Inflammatory Bowel Disease Patients: A Nationwide Population-Based Cohort Study

**DOI:** 10.3390/jcm15082972

**Published:** 2026-04-14

**Authors:** Seok-Hyung Won, Se-Heon Lee, Sung Hoon Jung, Jun-Seok Lee

**Affiliations:** 1 Department of Orthopedic Surgery, Eunpyeong St. Mary’s Hospital, College of Medicine, The Catholic University of Korea, Seoul 03312, Republic of Korea; markwon13@naver.com (S.-H.W.); dltpgjs202@naver.com (S.-H.L.); 2 Department of Internal Medicine, Eunpyeong St. Mary’s Hospital, College of Medicine, The Catholic University of Korea, Seoul 03312, Republic of Korea; shjung74@catholic.ac.kr

**Keywords:** femur fracture, inflammatory bowel disease, Crohn’s disease, ulcerative colitis, steroid

## Abstract

**Background/Objectives**: Patients with inflammatory bowel disease (IBD) have an increased fracture risk, but whether femur fracture risk differs from that of the general population remains unclear. We compared the risks of overall femur fractures and femur fractures requiring surgery in patients with IBD versus matched controls, and evaluated the association between corticosteroid exposure and femur fracture risk in IBD. **Methods**: Using the Korean National Health Insurance Service database, we identified patients newly diagnosed with Crohn’s disease (CD) or ulcerative colitis (UC) between 2008 and 2018. After 1:3 matching by age and sex, 33,778 patients with IBD (24,370 UC and 9408 CD) and 101,265 non-IBD controls were included. Incidence of femur fractures (overall and requiring surgery) was compared between groups. Multivariable analyses were performed to assess risk factors, including age, sex, disease subtype, comorbidity burden, and corticosteroid use duration. **Results**: Compared with controls, CD was associated with a higher incidence of femur fracture (HR, 2.10; 95% CI, 1.61–2.74; *p* < 0.001), whereas UC showed no clear association (HR, 1.15; 95% CI, 0.96–1.38; *p* = 0.120). In multivariable analysis, CD remained independently associated with femur fracture (HR, 1.74; 95% CI, 1.33–2.29; *p* < 0.001), while UC did not (HR, 0.99; 95% CI, 0.82–1.19; *p* = 0.890). Femur fractures requiring surgery were more frequent in both UC (HR, 1.35; 95% CI, 1.09–1.69; *p* = 0.028) and CD (HR, 1.67; 95% CI, 1.20–2.33; *p* = 0.003). Older age, female sex, higher comorbidity index, and longer corticosteroid exposure were associated with femur fracture in IBD. **Conclusions**: Only CD was associated with increased overall femur fracture risk; however, both CD and UC were associated with a higher risk of surgically treated femur fractures. Strategies to optimize bone health and minimize corticosteroid exposure may reduce femoral fracture risk in IBD.

## 1. Introduction

Inflammatory bowel disease (IBD) encompasses two debilitating chronic inflammatory diseases of the digestive tract, Crohn’s disease (CD) and ulcerative colitis (UC), that cause major suffering to patients and result in a high rate of disability, morbidity, and mortality [1]. Inflammatory bowel disease affects an estimated 0.3 to 1% of the worldwide population, and the incidence of IBD appears to be increasing substantially in the 21st century [2,3,4]. Both CD and UC can have several orthopedic manifestations, either because of the disease or due to treatment medications for the disease, causing alterations in bone mineral density and diminished overall bone health, ultimately leading to osteoporosis and a higher risk of fractures [5,6,7]. Such increased risk is not confined to fragility fractures (e.g., osteoporotic spine or hip fractures) but extends to fractures at various sites across a broader adult age range [8]. However, there is a lack of evidence focusing on the risk of overall femur fractures in this population.

Femur fractures are among the most serious musculoskeletal injuries and are associated with substantial short- and long-term morbidity in adults of all ages. Previous studies have shown that diaphyseal and proximal femoral fractures frequently occur in working-age adults, often following high-energy trauma, and typically require hospital admission, operative fixation, and prolonged rehabilitation [9], frequently leading to persistent pain, limitations in mobility, and loss of pre-injury level of function [10]. These injuries also impose a substantial socioeconomic burden: femoral fractures rank among the most expensive orthopedic injuries, with long hospital stays and high direct medical costs, as well as indirect productivity losses and out-of-pocket expenses for patients and society [11]. Accordingly, identification of the overall risk of femur fractures in patients with IBD, beyond osteoporotic hip fractures, is clinically relevant, particularly in those with prolonged exposure to systemic corticosteroids. This need is emphasized by the rising global prevalence of IBD, improved survival, and a trend toward younger age at diagnosis [12,13,14].

Accordingly, the primary aim of this nationwide, population-based cohort study is to compare the incidence of femur fractures in adults with IBD with the incidence of femur fractures in a reference population matched for age and sex. The secondary aim is to identify clinical factors associated with femur fractures and femur fractures requiring surgery in patients with IBD, with particular focus on comorbidity burden and the duration of systemic corticosteroid use.

## 2. Materials and Methods

Our nationwide, population-based cohort study compares the incidence of femur fractures in patients with IBD with the incidence of femur fractures in a non-IBD reference population in South Korea. The study protocol was approved by the Institutional Review Board of Eunpyeong St. Mary’s Hospital, The Catholic University of Korea (IRB approval number PC19ZNSE0128).

### 2.1. Data Source

We used the National Health Insurance Service (NHIS) database between 2008 and 2018. The database includes diagnostic codes based on the International Classification of Diseases, tenth revision (ICD-10), and special registration codes (V codes) from the Rare Intractable Diseases (RID) program. Because registration in the RID program requires confirmation of IBD based on clinical, endoscopic, and histologic findings, this system enabled accurate identification and characterization of patients with IBD.

### 2.2. Study Population

Patients with IBD were defined as those with both IBD-related ICD-10 diagnostic codes and corresponding RID V codes. Patients with CD were defined as the presence of an ICD-10 code K50~K50.9, together with RID code V130. Patients with UC were defined as an ICD-10 code K51~K51.9 together with RID code V131. A one-year washout period (2007) was applied to identify newly diagnosed patients. After this washout, 33,778 patients with IBD aged 18–79 years who were registered between 2008 and 2018 were included. The index date was defined as the first date on which both the ICD-10 and RID criteria for IBD were met. Patients with a history of femur fracture before the index date were excluded. For the non-IBD reference population, individuals without any IBD-related ICD-10 or RID codes were identified from the same NHIS database during the same period. A non-IBD reference population was selected at a 1:3 ratio to the IBD group using frequency matching by sex and 10-year age bands.

### 2.3. Outcomes

We collected information on age, sex, ICD-10 diagnostic codes, comorbidities, corticosteroid use, and IBD-related medications. Femur fractures were identified using ICD-10 codes S72.0, S72.1, S72.2, S72.3, S72.4, S72.7, S72.8, and S72.9, including open fracture codes associated with high-energy trauma (S72.061, S72.081, S72.091, S72.191, S72.21, S72.31, S72.71, S72.81, and S72.91). In addition, procedure codes for femur fracture surgery (N0601, N0611, N0711, N0715, N0991, N2070, and N2710) were extracted. The Charlson Comorbidity Index (CCI) was calculated to quantify baseline comorbidity burden and was categorized into four groups (0, 1, 2, and ≥3 points) according to the weighted score. Data on systemic corticosteroid use in patients with IBD were obtained from prescription records. The duration of corticosteroid use was categorized as <30 days, <90 days, <180 days, <365 days, and ≥1 year. Pertinent IBD-related medications included 5-aminosalicylic acid (5-ASA), immunomodulators (azathioprine, mercaptopurine, cyclosporine, tacrolimus, and methotrexate), corticosteroids, and biologic agents (infliximab, adalimumab, golimumab, and vedolizumab).

We compared cumulative events of femur fractures in IBD and non-IBD patients. In addition, we analyzed risk factors for femur fracture and compared cumulative events of femur fractures in UC and CD patients according to duration of steroid use. To focus on clinically meaningful events, we analyzed the incidence and risks of femur fractures requiring surgery. Patients who had both fracture diagnostic codes and corresponding surgical procedure codes were considered patients who experienced femur fractures requiring surgery.

### 2.4. Statistical Analysis

Descriptive statistics were used to summarize baseline characteristics of the IBD and reference groups. Differences between groups were assessed using chi-square tests for categorical variables and the Mann–Whitney U test for continuous variables. We assessed the cumulative femoral fracture incidence between IBD patients and the general population and performed Cox regression analyses to determine whether the duration of steroid use was associated with the occurrence of femoral fractures in IBD patients and the general population. The proportional hazards assumption was evaluated using Schoenfeld residuals. The multivariable model included age, sex, disease type (CD or UC), Charlson Comorbidity Index, and steroid exposure, which were selected based on clinical relevance and prior literature. Competing risks such as mortality were not explicitly modeled because the study population consisted predominantly of relatively young individuals with low mortality during follow-up. Hazard ratios (HRs) and 95% confidence intervals (CIs) were estimated. All statistical analyses were performed using R version 4.0.0 (R Foundation for Statistical Computing, Vienna, Austria) and SAS version 9.4 (SAS Institute Inc., Cary, NC, USA).

### 2.5. Declaration of Generative Artificial Intelligence (AI) in Scientific Writing

During the preparation of this work, the authors used ChatGPT (OpenAI; GPT-5.4, San Francisco, CA, USA) for language editing to improve clarity and grammar. After using this tool, the authors reviewed and revised the content as needed. The authors take full responsibility for the final version of the manuscript.

## 3. Results

### 3.1. Demographic Characteristics

From 2008 to 2018, the study population was 33,778 patients with IBD (24,370 UC patients and 9408 CD patients) and 101,265 patients in the reference population. Patient median age at IBD diagnosis was 39 years, and patients were followed up for a median of 5.0 years. The CD patients were younger and more likely to be male than the UC patients. The characteristics of subjects are listed in Table 1.

### 3.2. Cumulative Events of Femur Fractures

**Figure 1:**
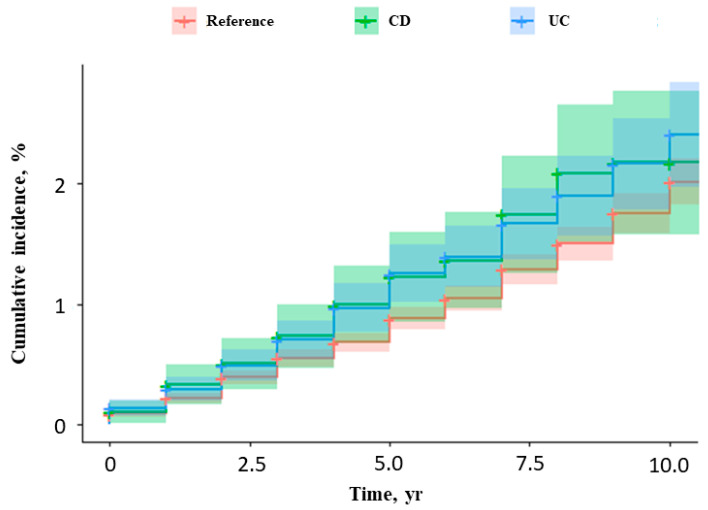
Cumulative incidence of femur fractures in inflammatory bowel disease patients and matched controls. *CD, Crohn’s disease; UC, ulcerative colitis*.

Among IBD patients, 217 femur fractures occurred during the study period (157 in UC patients and 60 in CD patients). In the reference population, 550 femur fractures occurred. During the follow-up period, the incidence rate ratio (IRR) of femur fractures in the IBD patients was 1.20 per 1000 person-years (95% confidence interval [CI], 1.04–1.37; *p* < 0.001) (Table 2). In comparison to the matched reference cohort, patients with CD had a significantly higher risk of femur fracture (hazard ratio [HR], 2.10; 95% CI, 1.61–2.74; *p* < 0.001), whereas patients with UC showed a non-significant tendency toward increased risk (HR, 1.15; 95% CI, 0.96–1.38; *p* = 0.120) (Figure 1, Table 3).

### 3.3. Risk Factors for Femur Fractures

In univariate analyses, UC patients tended to have a higher HR for femur fracture than the general population (HR, 1.26; 95% CI, 1.06–1.51; *p* < 0.001), but did not show significant differences in the multivariate analysis (HR, 0.99; 95% CI, 0.82–1.19; *p* = 0.890). In contrast, CD patients showed a higher risk of femur fracture than the general population (HR, 1.74; 95% CI, 1.33–2.29; *p* < 0.001) in the multivariate analysis, with no differences shown in UC patients (HR, 0.99; 95% CI, 0.82–1.19; *p* = 0.890). Older age, female sex, high CCI score, and longer duration of steroid use were significantly associated with femur fracture in both univariate and multivariate analyses (Table 4).

### 3.4. Cumulative Events of Femur Fracture in UC and CD Patients by Duration of Steroid Use

**Figure 2:**
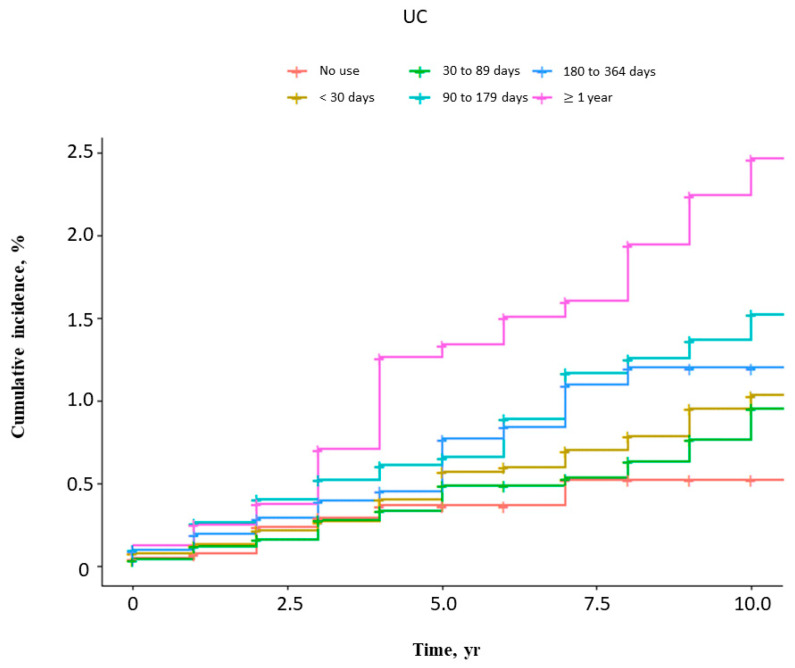
Cumulative incidence of femur fractures in UC patients stratified by duration of steroid use.

**Figure 3:**
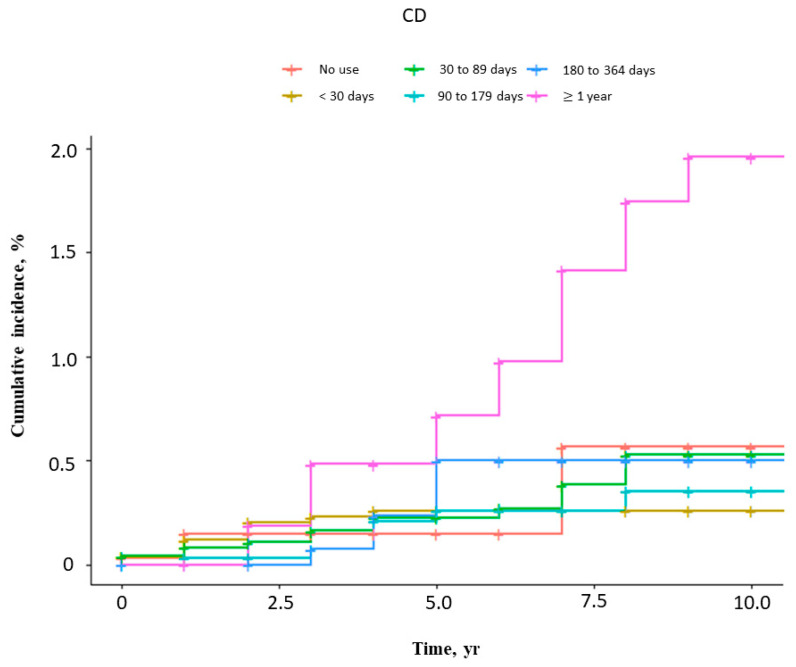
Cumulative incidence of femur fractures in CD patients stratified by duration of steroid use.

The risks of femur fractures in UC and CD patients by duration of steroid use are shown in Figure 2 and Figure 3 and Table 5. In both UC and CD patients, steroid exposure for less than one year was not significantly associated with risk, whereas exposure for one year or longer was independently associated with a higher risk of femur fracture.

## 4. Discussion

In our study, the incidence of femur fractures among IBD patients is higher than in the general population. When stratified by disease subtype, both CD and UC groups also show higher incidence rates than the reference population, with more than a twofold increase in risk among patients with CD, whereas patients with UC exhibit a non-significant tendency toward increased risk. Such results are consistent with previous large cohort studies of Western and Asian populations with IBD [15,16,17,18,19]. However, most prior studies have examined overall osteoporotic fractures or have focused specifically on hip and vertebral fractures, rather than evaluating femur fractures across the broader adult age range. Given that proximal and diaphyseal femoral fractures have been associated with higher rates of medical complications and mortality [20,21], our findings are clinically relevant in that they suggest an increased risk of femoral fractures as a whole, rather than fragility hip fractures alone, among patients with IBD.

In our multivariate analysis, the risk of femur fracture is higher in patients with CD than in UC patients, with only the CD group showing a significantly increased HR in comparison to the general population. This pattern is consistent with previous reports indicating that hip fractures and any fractures occur more frequently in CD than in UC [15,17]. In a Swedish nationwide cohort of 83,435 patients with IBD, the relative risks of hip fracture were 1.7 in CD and 1.3 in UC, whereas the relative risks of any fracture were 1.2 in both groups [17]. Similarly, a recent population-based study of 18,228 patients with IBD from South Korea reports incidence rate ratios for fractures of 1.15 and 0.96 in CD and UC, respectively, among patients diagnosed before 60 years of age, and 2.19 and 1.32, respectively, among those diagnosed at ≥60 years of age [15]. Although reasons for the higher fracture risk in CD in comparison to UC are not fully understood, they may relate to differences in age at disease onset, disease location and severity, inflammatory activity, and the profile of inflammatory mediators in patients [22,23].

Compared with the matched reference population, both CD and UC patients in our cohort are shown to have a significantly higher risk of femur fractures requiring surgical treatment. This contrasts with the pattern observed for overall femur fractures, in which only CD patients show a significantly increased risk, with a non-significant trend showing in UC patients. Most femur fractures in adults are treated with major orthopedic procedures, including hip arthroplasty or osteosynthesis using intramedullary nails or plates. Large database studies and meta-analyses have shown that patients with IBD undergoing joint arthroplasty are at higher risk of postoperative medical complications, infection, readmission, and implant revision than patients without IBD [24,25]. In addition, a Medicare-based cohort of hip fracture hospitalizations, in which almost 90% of patients underwent surgical treatment, reported longer length of stay and higher readmission rates among patients with IBD than among matched controls [26]. The treatment outcomes for femoral fractures in IBD patients are worse than those of non-IBD patients. Therefore, our results suggest that it is important to prevent femur fractures in IBD patients. Especially, IBD patients who are older, female, and who have high CCI scores and longer durations of steroid use are shown to be significantly associated with femur fracture. Thus, additional efforts may be needed to prevent and treat osteoporosis and to provide fall-prevention education for patients with these characteristics.

Systemic corticosteroids are a major cause of secondary osteoporosis and fracture, and patients with IBD frequently require repeated or prolonged courses of steroid therapy, warranting particular caution with respect to bone quality [27]. In a recent meta-analysis including 470,541 patients with IBD, those with fractures were more likely to be receiving corticosteroids than those without fractures (odds ratio, 1.47) [28]. Several large-scale studies have also shown that higher cumulative steroid exposure is associated with an increased risk of fractures in IBD [29]. In our study, the risk of femur fracture in both UC and CD is shown to be related to long-term steroid use, and this association is evident after ≥1 year of exposure. This one-year threshold can serve as a practical reference for determining the benefits of steroid treatment, considering the increased risk of femoral fractures in IBD patients.

Our study has several limitations. First, bone mineral density is not evaluated in the study cohort because bone densitometry data are not available in the NHIS claims database. Such a lack of data on bone mineral density may be an inevitable limitation of big data research. Previous studies have reported that low bone mineral density and osteoporosis may be more common in patients with CD than in those with UC, although the relative contribution of disease subtype and corticosteroid exposure remains inconsistent across studies. Adjustment for bone mineral density could potentially modify observed associations. Second, data on medical history, such as anemia, hypertension, diabetes, osteoporosis, and other metabolic diseases that may affect a patient’s disease and femur fracture, are limited and cannot be analyzed herein. In addition, individual frailty and fall risk are not assessable in the claims data used for our analyses. Although smoking status, alcohol consumption, and body mass index may be available in linked NHIS health screening data, these lifestyle factors are not included in our study. Third, because this study is based on retrospective data from the NHIS claims database, fractures occurring after IBD diagnosis are not distinguished from additional fractures on the contralateral side. Our analysis is therefore restricted to the first femur fracture after IBD diagnosis, and consequent femoral refractures are not evaluated. Fourth, steroid exposure in patients is assessed based on duration rather than cumulative dose; accordingly, a true dose–response relationship between cumulative steroid dose and fracture risk could not be evaluated. Prior studies have shown that both cumulative and daily corticosteroid doses are associated with an increased risk of osteoporotic fractures. Because detailed information regarding cumulative steroid dose or intermittent treatment patterns is not available in the NHIS database, our analysis uses steroid exposure duration as a surrogate measure. Further large-scale studies incorporating cumulative steroid dose are warranted to better clarify the association between steroid exposure and fracture risk in IBD patients. Fifth, the present analysis does not include lifestyle factors such as physical activity and smoking, and detailed nutritional factors such as vitamin D or calcium intake are not available in the dataset used. Given that patients with IBD may have impaired vitamin D and calcium metabolism, the potential influence of these factors on fracture risk is not accounted for in the present study. Sixth, steroid exposure is categorized according to cumulative duration and included as a fixed covariate in the Cox regression model rather than modeled as a time-varying variable. Therefore, the possibility of immortal time bias cannot be excluded, which may have resulted in underestimation of the fracture risk associated with longer steroid exposure, particularly in patients with steroid exposure of ≥1 year. Seventh, the NHIS claims database does not provide sufficiently detailed clinical information on IBD severity, anatomical disease location, or prior disease-related complications. Therefore, we could not assess whether differences in these disease characteristics between Crohn’s disease and ulcerative colitis contributed to the observed differences in femur fracture risk, and residual confounding by underlying disease burden cannot be excluded. Finally, although we evaluated femoral fractures as a whole, we did not perform subgroup analyses by anatomical location (e.g., femoral neck, intertrochanteric, subtrochanteric, or shaft). Accordingly, site-specific incidence is not reported. Furthermore, because the NHIS claims database does not provide detailed information regarding injury mechanism, fragility fractures are not distinguished from high-energy traumatic fractures. Although external cause codes exist in the NHIS database, they are incompletely recorded and do not reliably distinguish high-energy trauma from low-energy fragility fractures. Therefore, the present study does not differentiate the fracture mechanism. Consequently, our analysis includes both types of femur fractures, which may have influenced the interpretation of fracture risk related to bone health.

## 5. Conclusions

The incidence of femur fractures is shown to be higher in patients with IBD than in the general population. When analyzed by disease subtype, patients with CD are shown to have a significantly increased risk of femur fracture, whereas those with UC show only a non-significant tendency toward higher risk. In contrast, the risk of femur fractures requiring surgical treatment is significantly higher than in the general population in both CD and UC. Additionally, steroid use for ≥1 year is shown to be associated with an increased risk of femur fracture in both CD and UC. Clinicians should recognize this elevated fracture risk in the management of IBD and ensure appropriate surveillance and osteoporosis prevention, including regular bone mineral density monitoring and supplementation with vitamin D. Particular attention should be given to IBD patients who receive long-term systemic corticosteroid therapy, especially those treated for one year or longer.

## Figures and Tables

**Table 1:** Baseline characteristics of the study population, N (%).

Variables	General Population (*n*,%)	IBD (*n*,%)	CD (*n*,%)	UC (*n*,%)
	(N = 101,265)	(N = 33,778)	(N = 9408)	(N = 24,370)
**Age at diagnosis**				
**Years, median [IQR]**	39.0 [27.0; 52.0]	39.0 [27.0; 52.0]	28.0 [21.0; 42.0]	43.0 [31.0; 54.0]
**Sex, male**	62,625 (61.4%)	20,907 (61.4%)	6514 (68.8%)	14,393 (58.6%)
**Femur fracture**	550 (0.5%)	217 (0.6%)	60 (0.6%)	157 (0.6%)
**Surgery**				
**No**	184 (0.2%)	59 (0.2%)	16 (0.2%)	43 (0.2%)
**Yes**	366 (0.4%)	158 (0.5%)	44 (0.5%)	114 (0.5%)
**CCI group**				
**0**	60,620 (59.4%)	14,771 (43.4%)	3909 (41.3%)	10,862 (44.2%)
**1**	21,948 (21.5%)	9854 (29.0%)	2922 (30.9%)	6932 (28.2%)
**2**	9571 (9.4%)	5000 (14.7%)	1499 (15.8%)	3501 (14.3%)
**3**	9895 (9.7%)	4407 (12.9%)	1141 (12.0%)	3266 (13.3%)
**Use of medication**				
**5-ASA**		27,801 (81.7%)	8133 (85.9%)	19,668 (80.1%)
**Immune suppressors**		10,619 (31.2%)	6033 (63.7%)	4586 (18.7%)
**Biologics**		4088 (12.0%)	2329 (24.6%)	1759 (7.2%)
**Use of steroids**				
**<30 days**	51,530 (50.5%)	10,584 (31.1%)	2622 (27.7%)	7962 (32.4%)
**<90 days**	19,290 (18.9%)	7800 (22.9%)	2492 (26.3%)	5308 (21.6%)
**<180 days**	5717 (5.6%)	4611 (13.5%)	1521 (16.1%)	3090 (12.6%)
**<365 days**	2220 (2.2%)	2873 (8.4%)	759 (8.0%)	2114 (8.6%)
**≥1 year**	1135 (1.1%)	2148 (6.3%)	548 (5.8%)	1600 (6.5%)
**Follow-up duration**				
**Years, median [IQR]**	5.0 [3.0; 9.0]	5.0 [2.0; 8.0]	5.0 [2.0; 8.0]	5.0 [2.0; 8.0]

*IBD, inflammatory bowel disease; CD, Crohn’s disease; UC, ulcerative colitis; IQR, interquartile range; CCI, Charlson Comorbidity Index; 5-ASA, 5-aminosalicylic acid.*

**Table 2:** Incidence of femur fracture.

	Observed Cases	Sum of Person-Years	Incidence Rate/ 1000 Person-Years	95%CI	Incidence Rate Ratio	95%CI	*p*-Value
**General**	550	582,710	0.94	0.87–1.03	1.00		
**IBD**	217	181,212	1.2	1.04–1.37	1.27	1.26–1.28	<0.001
**CD**	60	50,436	1.19	1.01–1.53	1.26	1.25–1.27	<0.001
**UC**	157	130,776	1.2	1.02–1.40	1.27	1.26–1.28	<0.001

*IBD, inflammatory bowel disease; CD, Crohn’s disease; UC, ulcerative colitis.*

**Table 3:** Hazard ratios for femur fractures.

Variables	HR	95% CI	*p*-Value
**Disease entity**			
**General population**	1	-	-
**CD**	2.10	1.61–2.74	<0.001
**UC**	1.15	0.96–1.38	0.120
**Age**			
**Years**	1.09	1.08–1.09	<0.001
**Sex**			
**Male**	1	-	-
**Female**	1.31	1.11–1.85	<0.001

*CD, Crohn’s disease; UC, ulcerative colitis.*

**Table 4:** Risk factors of femur fracture.

		Univariate Analysis				Multivariate Analysis			
		HR	Lower 95	Upper 95	*p*-Value	HR	Lower 95	Upper 95	*p*-Value
**Disease**	**General**	1.00	-	-	-	1.00	-	-	-
	**CD**	1.25	0.96	1.64	0.100	1.74	1.33	2.29	<0.001
	**UC**	1.26	1.06	1.51	<0.001	0.99	0.82	1.19	0.891
**Age**		1.09	1.08	1.09	<0.001	1.08	1.07	1.08	<0.001
**Sex**	**Male**	1.00	-	-	-	1.00	-	-	-
	**Female**	1.64	1.42	1.89	<0.001	1.28	1.11	1.47	<0.001
**Steroid use**	**0**	1.00	-	-	-	1.00	-	-	-
	**<30 days**	0.96	0.76	1.22	0.762	0.92	0.73	1.16	0.470
	**<90 days**	1.00	0.78	1.30	0.971	0.85	0.65	1.10	0.222
	**<180 days**	1.40	1.04	1.88	<0.001	1.06	0.78	1.43	0.713
	**<365 days**	1.88	1.35	2.61	<0.001	1.31	0.94	1.83	0.110
	**≥1 year**	3.72	2.75	5.04	<0.001	2.13	1.56	2.91	<0.001
**CCI**	**0**	1.00	-	-	-	1.00	-	-	-
	**1**	1.47	1.20	1.81	<0.001	0.99	0.80	1.22	0.910
	**2**	2.30	1.83	2.89	<0.001	1.09	0.86	1.38	0.490
	**3**	6.23	5.24	7.41	<0.001	1.87	1.54	2.26	<0.001

*CD, Crohn’s disease; UC, ulcerative colitis; CCI, Charlson Comorbidity Index.*

**Table 5:** Hazard Ratio for femur fracture in CD and UC patients.

Variables	CD			UC		
	HR	95% CI	*p*-Value	HR	95% CI	*p*-Value
Age						
Years	1.08	1.06–1.09	<0.001	1.08	1.07–1.10	<0.001
Sex						
Male	1	-	-	1	-	-
Female	1.20	0.70–2.03	0.510	1.32	0.96–1.80	0.091
Steroid use						
0	1	-	-	1	-	-
<30 days	0.56	0.21–1.50	0.253	1.34	0.67–2.68	0.400
<90 days	0.92	0.35–2.42	0.874	1.12	0.54–2.32	0.772
<180 days	0.79	0.26–2.36	0.676	1.96	0.95–4.04	0.077
<365 days	1.22	0.39–2.36	0.741	1.73	0.80–3.73	0.164
≥1 year	2.62	1.00–6.86	<0.05	2.83	1.37–5.84	<0.001

## Data Availability

Data analyzed in this study were obtained from the Korean National Health Insurance Service (NHIS) database. Restrictions apply to the availability of these data, which were used under license for the current study. Therefore, the datasets are not publicly available due to legal and confidentiality requirements, but may be accessed through the National Health Insurance Sharing Service (NHISS) by researchers who meet criteria for access to confidential data.

## Abbreviations

The following abbreviations are used in this manuscript:

IBD	Inflammatory bowel disease
CD	Crohn’s disease
UC	Ulcerative colitis
NHIS	National Healthcare Insurance Service
CCI	Charlson Comorbidity Index
5-ASA	5-aminosalicylic acid
